# Unrelated cord blood transplantation vs. HLA-matched sibling transplantation for adults with B-cell acute lymphoblastic leukemia in complete remission: superior OS for patients with long-term survival

**DOI:** 10.1186/s13287-022-03186-3

**Published:** 2022-10-09

**Authors:** Guangyu Sun, Baolin Tang, Kaidi Song, Yue Wu, Meijuan Tu, Xiang Wan, Wen Yao, Liangquan Geng, Ping Qiang, Xiaoyu Zhu

**Affiliations:** 1 Department of Hematology, The First Affiliated Hospital of University of Science and Technology of China, Division of Life Sciences and Medicine, University of Science and Technology of China, Hefei, 230001 Anhui China; 2 Anhui Provincial Key Laboratory of Blood Research and Applications, Hefei, China; 3 Blood and Cell Therapy Institute, Division of Life Sciences and Medicine, University of Science and Technology of China, Hefei, China

**Keywords:** Acute lymphoblastic leukemia, Hematopoietic stem cell transplantation, Unrelated cord blood transplantation, Matched sibling transplantation

## Abstract

**Background:**

Allogeneic hematopoietic stem cell transplantation (allo-HSCT) is an important curative therapy for adult acute lymphoblastic leukemia (ALL). For patients who lack a human leukocyte antigen (HLA)-matched sibling donor, unrelated cord blood (UCB) is an alternative graft option. Previous studies have focused mainly on all T- and B-cell ALL (B-ALL) patients, while data related specifically to adult B-ALL patients after UCB transplantation (UCBT) are scarce.

**Methods:**

We retrospectively compared the outcomes of UCBT and HLA-matched sibling transplantation (MST) in the treatment of adult B-ALL patients in complete remission (CR) at our center. From June 2006 to December 2020, 156 adult B-ALL patients who achieved CR before transplantation were enrolled. The main clinical outcomes of UCBT and MST were analyzed.

**Results:**

Hematopoietic recovery was significantly faster in MST recipients than in UCBT recipients. Higher incidences of grades II-IV and III-IV acute graft-versus host disease (aGVHD) were found in UCBT recipients (*P* < 0.001 and = 0.03), while a lower incidence of extensive chronic GVHD (cGVHD) was found in UCBT recipients (*P* < 0.001). The cumulative incidences of 2-year non-relapse mortality (NRM), 2-year relapse, 5-year disease-free survival (DFS) and 5-year GVHD-free relapse-free survival (GRFS) were comparable between MST and UCBT recipients. The overall survival (OS) during the first 700 days was similar between the MST and UCBT groups, while the OS of patients with a survival time of more than 700 days in the UCBT group was better than that in the MST group according to multivariate analysis (*P* = 0.03).

**Conclusions:**

Our study shows that when treating adult B-ALL patients in CR, UCBT can achieve comparable effects as MST, may provide superior OS for patients with long-term survival, and should be considered a good alternative.

**Supplementary Information:**

The online version contains supplementary material available at 10.1186/s13287-022-03186-3.

## Introduction

Although remission can be attained in the majority of adult ALL patients through standard remission-induction chemotherapy, the risk of relapse still remains high, with a remission rate that varies from 30 to 80% [1, 2]. Allo-HSCT is an important curative therapy for adult ALL. Allo-HSCT provide superior disease-free survival (DFS) and overall survival (OS) for both standard-risk and high-risk adult ALL patients comparing with chemotherapy or autologous hematopoietic stem cell transplant [3–6]. For adult ALL patients, MST is the optimal option [7]. However, only 25% to 30% of patients are able to find a suitable related donor, and 40% to 50% of white patients who fail to obtain an HLA-identical sibling will find a matched unrelated donor [8]. For other patients, alternative donors, including mismatched unrelated donors, UCB, or a haploidentical donor, can be considered [8].

In the era of cellular immunotherapy, therapeutic strategies of B-ALL are distinct from those of T-cell ALL (T-ALL), as chimeric antigen receptor T-cell (CAR-T) therapy has shown excellent effect on B-ALL but limited effect on T-ALL [9]. Several studies have shown that CAR-T therapy followed by allo-HSCT can improve the prognosis of relapsed/refractory B-ALL (R/R B-ALL), in which hematopoietic stem cells come from HLA-matched siblings, haploidentical donors or UCB [10–12]. UCB is an alternative graft for patients who lack an HLA-matched donor. UCB possesses advantages including a lower incidence of cGVHD, less stringent HLA matching and rapid availability, which makes it suitable for nearly all patients [13], particularly for R/R B-ALL patients after CAR-T therapy who need urgent allo-HSCT.

UCBT has been used successfully among adult ALL patients [14–17]. Our previous study revealed that UCBT provided a similar OS as MST or haplo-HSCT for pediatric ALL [18, 19]. In the above studies, more attention was focused on the entire ALL patient population composed of B-ALL and T-ALL patients, while data specific to adult B-ALL patients after UCBT were scarce. Therefore, in the present study, we compared the outcomes of sUCBT and MST in the treatment of adult B-ALL in CR at our center.

## Methods

### Patients

**Fig. 1:**
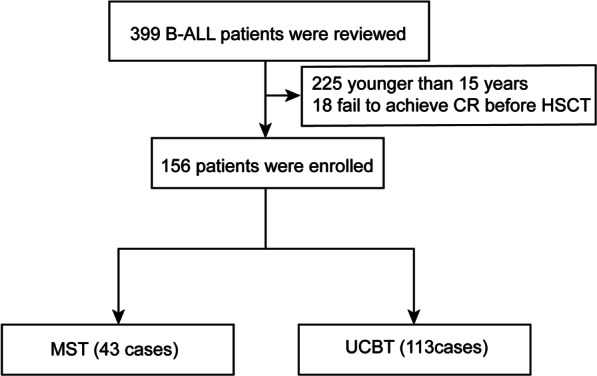
Flowchart of patient enrollment in the study

Between June 2006 and December 2020, 399 consecutive patients with B-ALL who received either UCBT or MST at our center were reviewed. The exclusion criteria were as follows: (1) patients who were younger than 15 years or older than 60 years when receiving allo-HSCT; (2) failing to achieve CR before transplantation; (3) patients receiving other types of allo-HSCT except UCBT and MST; (4) having a history of HSCT before. Finally, a total of 156 patients were enrolled in the final analysis. Among them, 43 patients (27.6%) received MST, and the remaining 113 patients (72.4%) received sUCBT (Fig. 1).

### Transplantation procedure

HLA-matched sibling donors were the preferred option for allo-HSCT. UCBT was performed when a suitable related donor or alternative unrelated donor was unavailable.

For UCBT recipients, cord blood selection and HLA typing followed the same procedures as those described previously [20]. In simple terms, HLA typing of cord blood and recipients was performed using molecular techniques with a minimum antigen split-level resolution for HLA-A and HLA-B and allele-level resolution for HLA-DRB1. Cord blood units obtained from Chinese cord blood banks were matched with a recipient for ≥ 4/6 HLA-A and HLA-B antigen, HLA-DRB1 high resolution. Cell counts should contain at least 3.0 × 10^7^/kg of recipient body weight total nucleated cells (TNCs) and 1.2 × 10^5^/kg CD34^+^ cells before freezing.

For UCBT recipients, the conditioning regimen consisted of intravenous (IV) busulfan (BU, total 12.8 mg/kg, 0.8 mg/kg every 6 h for 4 days), cyclophosphamide (CY, 60 mg/kg daily for 2 days) (BU/CY) plus fludarabine (FLU, 30 mg/m^2^ daily for 4 days) (*n* = 70, 61.9%), total body irradiation (TBI, total 12 Gy in 4 fractions) and CY (60 mg/kg daily for 2 days) (TBI/CY) plus cytarabine (2 g/m^2^ twice for 2 days) (*n* = 43,38.1%). A combination of cyclosporin A (CsA) and mycophenolate mofetil (MMF) was used to prevent GVHD for all UCBT recipients except one patient (0.9%) who received extra short-term methotrexate (MTX). MST recipients received a myeloablative conditioning regimen including BU/CY only (*n* = 3,7.0%) or plus FLU (*n* = 19, 44.2%) or TBI/CY (*n* = 21,48.8%). GVHD prophylaxis comprised CsA and MMF with or without short-term MTX (5 cases, 11.6%; and 38 cases, 88.4%, respectively).

### Definitions

CR was defined as the presence of < 5% blasts in the BM without EMD. CR1 was defined as achieving CR after induction therapy and continued without disease relapse, and CR2 was defined as achieving the second CR after experiencing the first recurrence of ALL. Real-time quantitative polymerase chain reaction (RT–qPCR) with a sensitivity of 10^–4^ ABL was used to evaluate the minimal residual disease (MRD) status for patients harboring BCR/ABL fusion gene, while multi-parametric flow cytometry with a sensitivity of 10^–4^ nucleated cells was used for the remaining patients (Additional file 1: Data). Induction failure was defined as failing to achieve CR after one cycle of induction chemotherapy.

Neutrophil engraftment was defined as the first of 3 consecutive days with absolute neutrophil count > 0.5 × 10^9^/L. Platelet engraftment was defined as the first of 7 consecutive days with a platelet count > 20 × 10^9^/L without transfusion support. The diagnosis and evaluation of aGVHD and cGVHD were in line with the commonly accepted criteria [21, 22]. OS was measured from the date of transplant until death from any cause or last follow-up. DFS was defined as survival in CR after transplantation. Relapse and death from any cause were treated as events. NRM was defined as death after transplantation without prior relapse. Relapse mortality (RM) was defined as death after HSCT with relapse. GRFS was defined as the absence of grade III-IV aGVHD, extensive cGVHD, relapse and death.

### Statistical analysis

Categorical variables were compared using the chi-square test and Fisher’s exact test. For continuous variables, the Mann–Whitney U test was used to compare differences. OS, DFS and GRFS were analyzed by the Kaplan–Meier method, while follow-up time was estimated using the reverse Kaplan–Meier method. Competing-risk analysis was used to evaluate the cumulative incidence of neutrophil and platelet engraftment, GVHD, NRM, RM and relapse. The Gray test was used to assess the differences between the MST and UCBT groups. Univariate and multivariate analyses of factors associated with NRM, relapse, OS and DFS were performed using a Cox proportional hazards model. Scaled Schoenfeld residuals were used to verify the proportional hazard (PH) assumption, and landmark analysis was applied if the PH assumption was violated [23]. The multivariate hazard ratios (HRs) for relapse and NRM were estimated from competing-risk regression analyses [23]. The factors with *P* < 0.20 in the univariate analysis were included in the multivariate regression analysis, and those with *P* < 0.05 in the multivariate analysis were considered significant. Data were analyzed using EZR version 1.55 [24], and all reported *P* values < 0.05 (2-sided) were considered to be statistically significant.

## Results

### Patient characteristics

All surviving patients were followed up until December 1, 2021, and the median follow-up time was 47.1 months (range, 8.9 to 181.8 months). The median age at the time of transplantation was 26 years (range, 15 to 58 years). There was no significant difference in the body weight of recipients at the time of transplantation between the UCBT and MST groups (*P* = 0.26). The demographic and basic characteristics of the patients are shown in Table 1. Patients who received UCBT were younger than patients who received MST (*P* = 0.002). Only a fraction of recipients (3.5%) were HLA-matched to donors (cord blood grafts) at HLA-A, B and DRB1 loci in the UCBT group, while the rest were HLA-mismatched. Compared with the MST group, the levels of infused TNCs and CD34^+^ cells were much lower in the UCBT group (both *P* values < 0.001). Seven patients (7.2%) achieved CR through CAR-T therapy before receiving UCBT, and all patients achieved CR through chemotherapy in the MST group. There were no significant differences with respect to ABO incompatibility, donor recipient sex matching, conditioning regimen, cytogenetic aberrations or MRD-positive frequency between the UCBT and MST groups.

**Table 1:** Patient characteristics between MST and UCBT group

Characteristics	UCBT(*n* = 113)	MST(*n* = 43)	*P*
Sex, male (%)	65 (57.5)	20 (46.5)	0.28
Age (years), Median (Range)	25 (15–57)	34 (15–58)	0.002
Weight (kg), Median (Range)	60 (40–93)	62 (36–83.6)	0.26
ABO Incompatibility, *n* (%)			0.14
Identical or minor incompatibility	68(60.2)	32 (74.4)	
Major or bidirectional incompatibility	45 (39.8)	11(25.6)	
HLA matching (/6), *n* (%)			< 0.001
4	62 (54.9)	0 (0.0)	
5	47 (41.6)	0 (0.0)	
6	4 (3.5)	43 (100.0)	
Donor/Receptor			1
F/M	28 (24.8)	10 (23.3)	
Others	85 (75.2)	33 (76.7)	
Conditioning (%)			0.28
BU/CY2 based	70 (61.9)	22 (51.2)	
TBI/CY based	43 (38.1)	21 (48.8)	
Infusion TNC, Median (Range,10^7^/Kg)	2.7 (1.0–6.8)	74.4 (24.6–160.0)	< 0.001
Infusion CD34^+^, Median (Range,10^5^/Kg)	1.8 (0.1–7.8)	4.2 (1.4–57.8)	< 0.001
Disease status before HSCT			0.25
CR1	75 (66.4)	33 (76.7)	
≥ CR2	38 (33.6)	10 (23.3)	
Cytogenetics			0.36
Normal, *n* (%)	53 (46.9)	21 (48.8)	
BCR/ABL, *n* (%)	40 (35.4)	20 (46.5)	
t(v;14q32), *n* (%)	5 (4.4)	1 (2.3)	
Complex karyotype, *n* (%)	3 (2.7)	0 (0.0)	
E2A/PBX1, *n* (%)	6 (5.3)	0 (0.0)	
HOX11 overexpression, *n* (%)	1 (0.9)	1 (2.3)	
Ph-like, *n* (%)	5 (4.4)	0 (0.0)	
Cytogenetical Prognosis, *n* (%)			0.86
Good^a^	60 (53.1)	22 (51.2)	
Poor^b^	53 (46.9)	21 (48.8)	
Received CAR-T before HSCT, *n* (%)	7 (6.2)	0 (0.0)	0.19
Transplantation time			0.05
2001–2016	42 (37.2)	24 (55.8)	
2017–2020	71 (62.8)	19 (44.2)	
GVHD prophylaxis, *n* (%)			0.006
CsA + MMF	112 (99.1)	38 (88.4)	
CsA + MMF + MTX	1 (0.9)	5 (11.6)	
MRD			0.69
Negative, *n* (%)	108 (95.6)	40 (93.0)	
Positive, *n* (%)	5 (4.4)	3 (7.0)	
Induction failure, *n* (%)	9 (8.0)	2 (4.7)	0.73
WBC at diagnosis			0.05
≤ 30 × 10^9^/L	76 (67.3)	36 (83.7)	
> 30 × 10^9^/L	37 (32.7)	7 (16.3)	

^a^Good risk cytogenetics included normal cytogenetics, HOX11 overexpression and *t* (12;21) (p13; q22): ETV6-RUNX1

^b^Poor risk cytogenetics included *t*(v;14q23)/IgH rearrangement, *t* (9;22) (q34; q11.2): BCR-ABL1, complex karyotype and Philadelphia chromosome-like

*BU* Busulfan, *CAR-T* Chimeric antigen receptor T-cell, *CR* Complete remission, *CsA* Cyclosporin A, *CY* Cyclophosphamide, *GVHD* Graft-versus-host disease, *HSCT* Hematopoietic stem cell transplant, *MMF* Mycophenolate mofetil, *MRD* Minimal residual disease, *MST* Matched sibling transplantation, *MTX* Methotrexate, *TBI* Total body irradiation, *TNC* Total nucleated cells, *UCBT* Unrelated cord blood transplantation, *WBC* White blood cell

### Engraftment of neutrophils and platelets

**Fig. 2:**
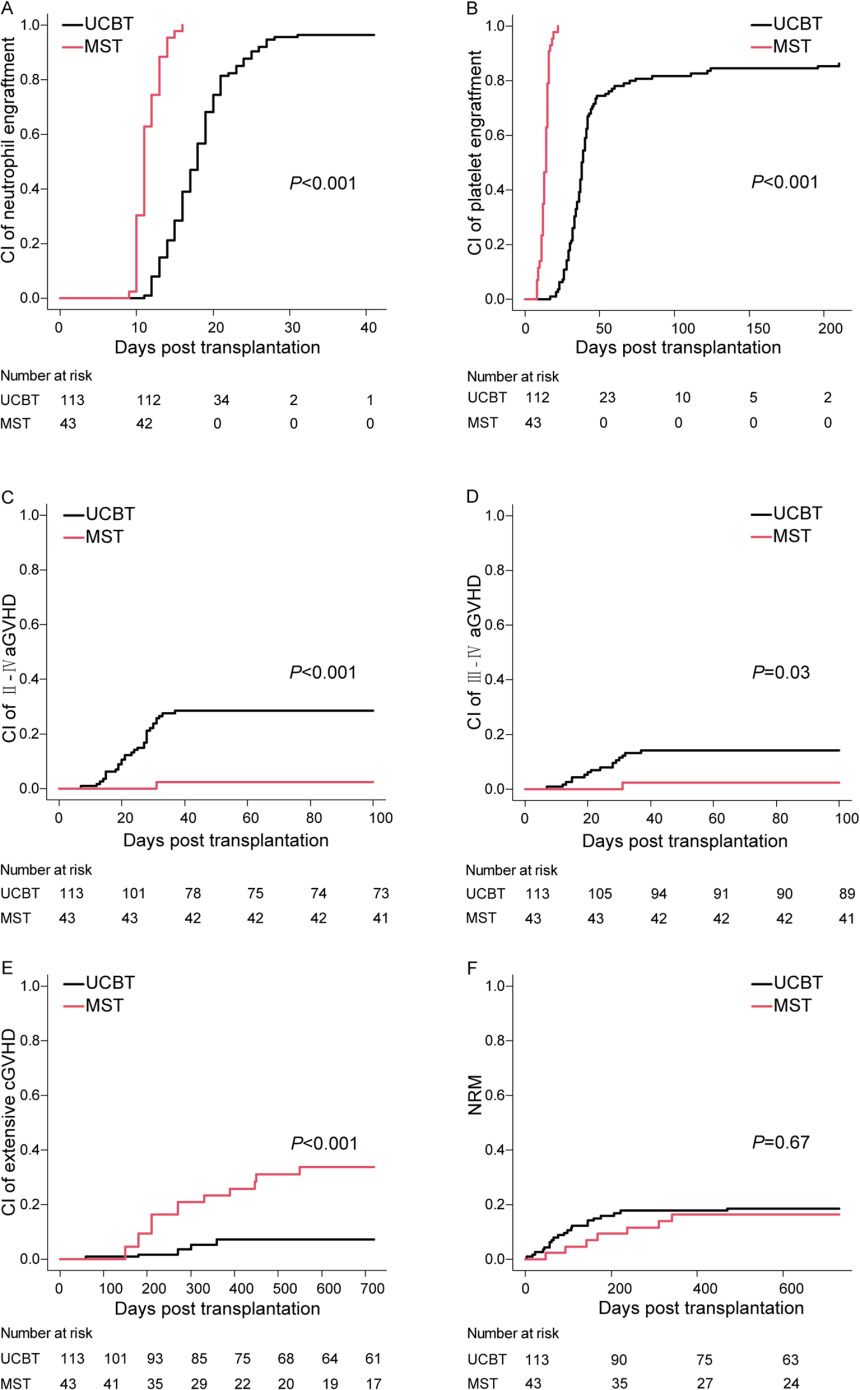
Cumulative incidence of neutrophil engraftment (**A)** platelet engraftment (**B**), II–IV aGVHD (**C**),III–IV aGVHD (**D**), extensive cGVHD (**E**) and NRM (**F**) in the UCBT and MST group

The cumulative incidence of neutrophil engraftment by day 42 was 96.5% (95% confidence interval [CI] 90.8–98.7%) in UCBT recipients and 100% in MST recipients (*P* < 0.001) (Fig. 2A). The median time of neutrophil engraftment was 18 (11–31) days in UCBT recipients and 11 (9–16) days in MST recipients. The cumulative incidence of platelet engraftment was 86.3% (95% CI 78.3–91.5%) in UCBT recipients and 100% in MST recipients (*P* < 0.001) (Fig. 2B). The median time of platelet engraftment was 38.5 (17–210) days in UCBT recipients and 14 (8–22) days in MST recipients.

### aGVHD and cGVHD

The cumulative incidence of grade II-IV aGVHD was 2.3% (95% CI 0.2–10.6%) in the MST group, which was much lower than that in the UCBT group (28.3%, 95% CI 20.4–36.8%, *P* < 0.001) (Fig. 2C). The cumulative incidence of grade III-IV aGVHD was also significantly lower in the MST group than in the UCBT group (2.3% [95% CI 0.2–10.6%] and 14.2% [95% CI, 8.5–21.2%], respectively, *P* = 0.03) (Fig. 2D). However, the 2-year cumulative incidence of extensive cGVHD in the MST group was significantly higher than that in the UCBT group [35.3% (95% CI, 20.9–50.0%) vs. 8.0% (95% CI, 3.7–14.3%), *P* < 0.001] (Fig. 2E).

### NRM, relapse and RM

The 2-year cumulative incidence of NRM was comparable between the MST and UCBT groups (MST, 16.3% [95% CI 7.2–28.7%], UCBT, 18.7% [95% CI 12.1–26.3%]) (*P* = 0.67) (Fig. 2F). The causes of NRM are summarized in Table 2. The 2-year cumulative incidence of relapse was also similar between the two groups: 17.0% (10.7–24.5%) in UCBT and 23.8% (12.3–37.4%) in MST (*P* = 0.35). The median time from transplantation to relapse was 9.0 months (4.1–16.5 months) in the UCBT group and 8.6 months (2.5–29.5 months) in the MST group. Eleven patients in the MST group and 19 patients in the UCBT group had suffered relapses at the last follow-up. In the MST group, three patients experienced extramedullary marrow relapses (EMRs), and eight experienced bone marrow relapses (BMRs). In the UCBT group, six patients experienced EMR, and thirteen experienced BMR. In the MST group, two patients received intrathecal injections, three received chemotherapies (one underwent a second HSCT), one received interferon therapy, two received palliative therapy and three received autologous CD19-targeted CAR-T therapy. In the UCBT group, two patients received intrathecal injections, two received radiotherapies, one received interferon therapy, one received chemotherapy, six received palliative therapy and seven received autologous CD19-targeted CAR-T therapy. Eight patients in the MST group and nine in the UCBT group had died of relapse at the time of last follow-up. The 2-year cumulative incidence of RM was comparable between the MST and UCBT groups (MST, 12.3% [95% CI 4.5–24.3%], UCBT, 8.5% [95% CI 4.2–14.8%]) (*P* = 0.54).

**Table 2:** Causes of NRM

	UCBT (*n* = 113)	MST(*n* = 43)
Causes, *n* (%)		
Infection	9 (8.0)	5 (11.6)
GVHD	4 (3.5)	1 (2.3)
Cerebral hemorrhage	4 (3.5)	0
heart failure	1 (0.8)	0
Multi-organ failure	3 (2.7)	0
Total	21	6

*NRM* Non-relapse mortality, *GVHD* Graft-versus-host disease

### OS, DFS and GRFS

**Fig. 3:**
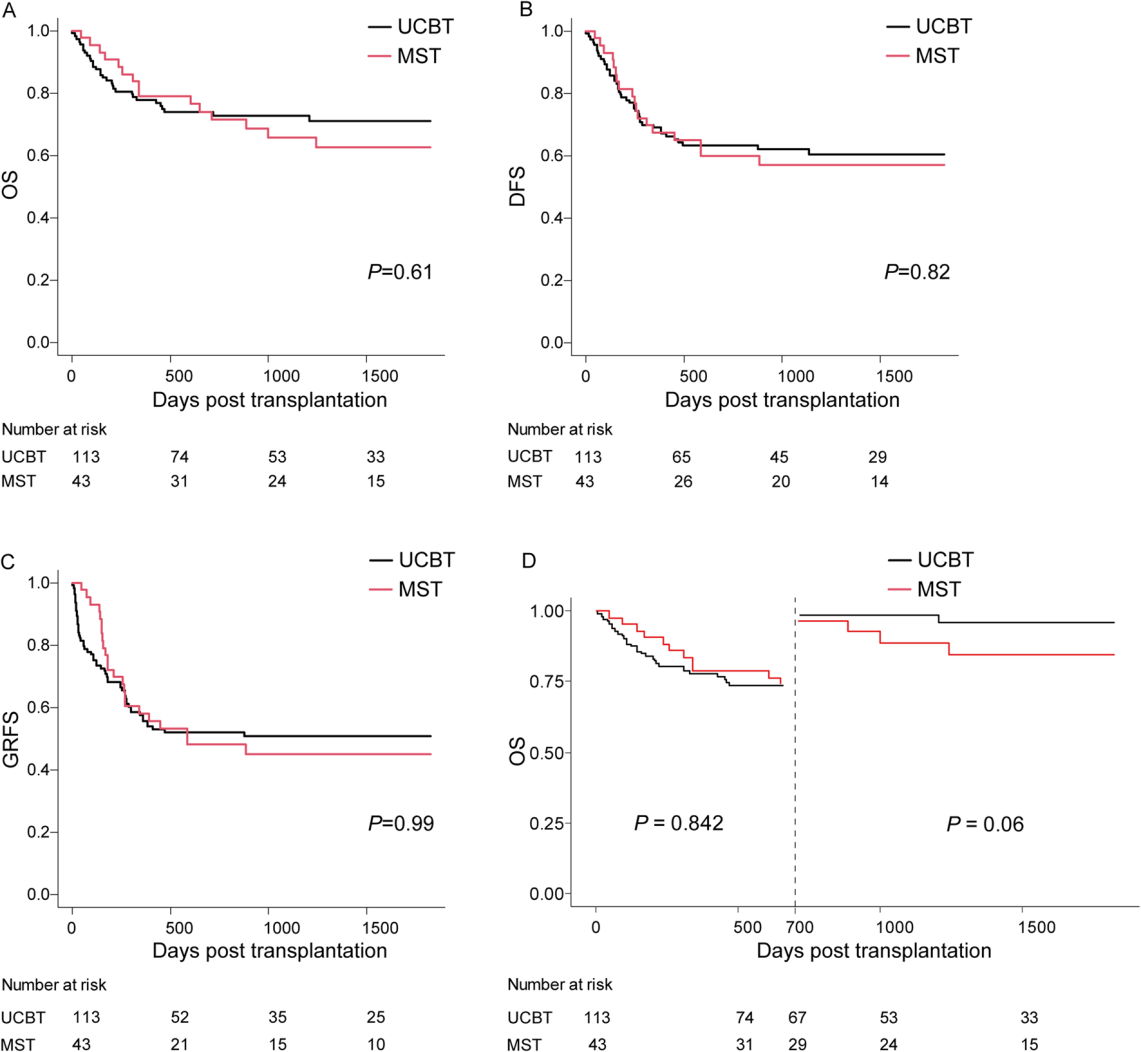
OS (**A**), DFS (**B**), GRFS (**C**) and landmark analysis of OS (**D**) between UCBT and MST group

The 5-year probabilities of OS in the MST and UCBT groups were 62.6% (95% CI 45.5–75.7%) and 71.1% (95% CI 61.3–78.9%), respectively (*P* = 0.61) (Fig. 3A). The probability of 5-year DFS in the MST group was comparable to that in the UCBT group (60.5%, 95% CI 50.3–69.2%; 57.1%, 95% CI 40.7–70.5%, *P* = 0.82) (Fig. 3B). The 5-year probabilities of GRFS in the MST and UCBT groups were 45.1% (95% CI 29.6–59.5%) and 50.8% (95% CI 41.1–59.6%), respectively (*P* = 0.99) (Fig. 3C).

As the variable ‘Group’ (UCBT vs. MST) violated the PH assumption, we performed landmark analysis to assess OS at 700 days and 700-day to 5-year post-transplantation. Univariate analysis indicated a similar OS between the MST and UCBT groups at 700-day post-transplantation (MTS: 74.0%, 95% CI 57.9–84.7%; UCBT: 73.9%, 95% CI 64.7–81.1%; *P* = 0.84). For patients who lived longer than 700 days, receiving UCBT was associated with superior OS compared with receiving MST (*P* = 0.06) (Fig. 3D), and multivariate analysis showed that receiving UCBT was an independent prognostic factor associated with better OS for patients who lived longer than 700 days (*P* = 0.03) (Table 3). Two patients died of relapse in the UCBT group, while three died of relapse and one died of pulmonary cGVHD in the MST group among patients who lived longer than 700 days.

**Table 3:** Uni- and multivariate analysis of the factors associated with transplantation outcome

Covariate	Univariate analysis			Multivariate analysis		
	HR	95%CI	*P*	HR	95%CI	*P*
OS > 700 days						
Conditioning BU/CY2 vs. TBI/CY	1.22	0.24–6.13	0.81			
MRD (N vs P)	8.11	0.84–78.08	0.07	1.00	0.08–12.18	0.97
Age (< 35y vs. ≥ 35y)	2.55	0.51–12.64	0.25			
Blood type Match & minor vs. major & bid	1.21	0.22–6.62	0.83			
F/M vs. Others	0.33	0.07–1.62	0.17	0.32	0.06–1.76	0.19
Transplantation time 2006–16 vs. 2017–20	0.55	0.10–3.06	0.49			
CR1 vs. ≥ CR2	6.61	1.21–36.19	0.03	8.32	1.30–53.11	0.03
WBC (× 10^9^/l) < 30 vs. ≥ 30	0.59	0.07–5.02	0.63			
Cytogenetical prognosis Good vs. poor	2.30	0.42–12.58	0.34			
UCBT vs MST	4.43	0.81–24.20	0.09	7.40	1.12–48.93	0.04
*DFS*						
Conditioning BU/CY2 vs. TBI/CY	1.86	1.13–3.11	0.02	1.99	1.00–3.95	0.05
MRD (N vs. P)	1.84	0.73–4.59	0.19	1.63	0.56–4.73	0.37
Age(< 35y vs ≥ 35y)	0.90	0.52–1.56	0.71			
Blood type Match & minor vs major & bid	1.56	0.94–2.60	0.09	1.64	0.96–2.80	0.07
F/M vs. others	0.93	0.53–1.65	0.81			
Transplantation time 2006–16 vs. 2017–20	0.68	0.41–1.13	0.13	0.87	0.43–1.75	0.70
Response to induction Good vs. poor	1.42	0.61–3.30	0.41			
CR1 vs. ≥ CR2	2.40	1.45–3.99	< 0.001	2.34	1.38–3.97	0.002
WBC (× 10^9^/l) < 30 vs. ≥ 30	1.16	0.67–2.00	0.61			
Cytogenetical prognosis Good vs. poor	0.77	0.46–1.28	0.31			
UCBT vs. MST	1.07	0.62–1.85	0.82	1.05	0.58–1.90	0.86

*BU* Busulfan, *CAR-T* Chimeric antigen receptor T-cell, *CR* Complete remission, *CsA* Cyclosporin A, *CY* Cyclophosphamide, *GVHD* Graft-versus-host disease, *HSCT* Hematopoietic stem cell transplant, *MMF* Mycophenolate mofetil, *MRD* Minimal residual disease, *MTX* Methotrexate, *TBI* Total body irradiation, *TNC* Total nucleated cells, *WBC* White blood cell

## Discussion

This study demonstrated that UCBT after myeloablative conditioning regimens resulted in equivalent long-term survival in comparison with MST for B-ALL, which was consistent with previous studies [17, 18, 25, 26]. Sharma et al. [17] discovered that compared with MST, double-unit UCBT could provide similar OS without higher transplantation related mortality (TRM) for adult hematological malignancies. However, the study included only 41 B-ALL patients and lacked comparative analysis between MST and UCBT. Comparable OS or DFS between MST and UCBT has been shown for pediatric acute leukemia patients in other studies [18, 25, 27]. For adult hematological malignancy, Takaaki et al. [28] found that patients who received sUCBT had higher TRM and inferior OS than patients with the same age who received MST, which was inconsistent with our results. Different from our study, patients included in the study were elderly patients (≥ 50 years), and higher early TRM was found in patients who received sUCBT comparing with MST, indicating that MST was still the best option for elderly patients. Similarly, designated comparison between UCBT and MTS for B-ALL was absent. As our knowledge, our study is the first study conducted specifically among adult B-ALL patients in CR that compared MST and sUCBT.

The use of UCB for allo-HSCT has the potential advantage of a lower risk of cGVHD [29]. Our results showed that the cumulative incidences of grades II-IV and III-IV aGVHD after UCBT were both significantly higher than those after MST. In our study, although almost all the patients (99.1%) who received UCBT and the majority of patients (88.4%) who received MST used the same GVHD prophylaxis regimen consisting of CsA plus MMF, HLA disparities were more common in the UCBT group than in the MST group, which may be the reason for the higher aGVHD incidence in the UCBT group [30]. Sharma P et al. [17] compared outcomes among adult patients receiving MST and double-unit UCBT, and the results showed that the cumulative incidence of grade II-IV aGVHD was higher among CBT patients than MST patients, whereas the cumulative incidence of grade III-IV aGVHD was comparable. However, the definite CI of grade III-IV aGVHD was not described in that study. Compared with previous studies [18, 31, 32], the CI of severe aGVHD in our study was not distinctively high. Consistent with previous outcomes [29, 33], our study showed that the CI of extensive cGVHD was significantly lower in the UCBT group than in the MST group.

NRM was higher, and hematopoietic recovery was lower after UCBT compared with after-matched BM and peripheral blood stem cell transplantation [15, 34, 35]. However, more recent studies found comparable NRM after UCBT compared with after MST and matched BM transplantation [17, 36]. In our study, although delayed hematopoietic recovery after UCBT was expected, UCBT showed noninferior NRM and OS compared with MST in adult B-ALL patients. In addition, superior OS was found in the population of patients who survived more than 700 days in our study after UCBT compared with MST. Another study involving pediatric acute leukemia patients failed to find superior long-term survival for UCBT recepients comparing with MST recepients, and comparable incidence of extensive cGVHD was found in that study [37]. Lower incidence of cGVHD after UCBT might contribute to reduced late mortality [38].

An enhanced graft-versus-leukemia effect after HLA-mismatched and T-cell-replete UCBT was observed through clinical or experimental data [18, 26, 39–42]. However, patients with hematological malignancies enrolled in these studies were either at high risk or in an advanced stage and MRD^+^, which represented a high disease burden among these cases. For patients still in CR, existing studies demonstrated that similar relapse rates were observed between UCBT and other types of transplantation [43–45]. In our study, all enrolled patients were in CR before transplantation. The results showed that the relapse rate after transplantation was comparable between UCBT and MST, indicating that matched sibling donors are still the preferred source of grafts for patients with a low disease burden. Since donor lymphocyte infusion may be an effective mean to decrease mortality among relapsed patients after transplantation, the inability to obtain donor lymphocytes when needed is one of the defects of UCBT [46]. However, a similar RM was found between UCBT and MST in our study, which was consistent with another study [19] comparing UCBT and haplo-HSCT. Noninferior survival rates for UCBT recipients after relapse may benefit from CAR-T therapy, as eligible patients received this effective treatment in our study.

Several studies [14, 19, 38, 43, 44] have reported comparable results between UCBT and haplo-HSCT. Among five studies, comparable DFS and lower incidences of cGVHD were reported by three [19, 38, 43]. Ruggeri et al. [38] analyzed a dataset including 528 adult ALL patients (sUCBT = 370 and haplo-HSCT = 158) and found no statistically significant differences in DFS after sUCBT and haplo-HSCT (HR = 1.00 and *P* = 0.84) but a higher cumulative incidence of cGVHD after haplo-HSCT (HR = 1.72 and *P* = 0.01). Similar results were also found in another study [19] conducted in our center and the Institute of Hematology, Peking University. With the improvement of GVHD prophylaxis after haplo-HSCT, such as the application of post-CY, the risk of cGVHD after haplo-HSCT has already decreased recently, and an equivalent incidence of cGVHD has been reported [14, 44]. Consequently, both UCB and haploidentical donors are valid for patients who need urgent HSCT and lack matched related donors, as both have the advantage of rapid availability.

## Conclusion

This study showed that compared with MST, sUCBT can provide noninferior clinical outcomes for adult B-ALL patients in CR and may provide superior OS among patients with long-term survival, which demonstrates that single-unit UCB is also suitable for adults in need. However, the limited sample size of patients receiving MST and the retrospective nature of the study indicate that our results need further support from large-scale, prospective studies.

## Supplementary Information

**Additional file 1: ** MRD assessment.

## Data Availability

The datasets of the current study are available from the corresponding author on reasonable request. All authors have accessed the database and verified its accuracy.

## Abbreviations

ALL: Acute lymphoblastic leukemia
aGVHD: Acute graft-versus-host disease
allo-HSCT: Allogeneic hematopoietic stem cell transplantation
B-ALL: B-cell ALL
BMRs: Bone marrow relapses
BU: Busulfan
CAR-T: Chimeric antigen receptor T-cell
cGVHD: Chronic GVHD
CI: Confidence interval
CR: Complete remission
CsA: Cyclosporin A
CY: Cyclophosphamide
DFS: Disease-free survival
EMRs: Extramedullary marrow relapses
FLU: Fludarabine
GRFS: GVHD-free relapse-free survival
haplo-HSCT: Haploidentical HSCT
HLA: Human leukocyte antigen
HRs: Hazard ratios
MMF: Mycophenolate mofetil
MRD: Minimal residual disease
MST: Matched sibling transplantation
MTX: Methotrexate
NRM: Non-relapse mortality
OS: Overall survival
PH: Proportional hazard
R/R B-ALL: Relapsed/refractory B-ALL
RM: Relapse mortality
RT–qPCR: Real-time quantitative polymerase chain reaction
sUCBT: Single-unit UCBT
T-ALL: T-cell ALL
TBI: Total body irradiation
TNCs: Total nucleated cells
TRM: Transplantation related mortality
UCB: Unrelated cord blood
UCBT: Unrelated cord blood transplantation
