# Supplementary material for: Unrelated cord blood transplantation vs. HLA-matched sibling transplantation for adults with B-cell acute lymphoblastic leukemia in complete remission: superior OS for patients with long-term survival
Source: Stem Cell Res Ther. 2022 Oct 9;13:500. doi: 10.1186/s13287-022-03186-3 (PMC9549614; doi:10.1186/s13287-022-03186-3)
Supplement: Supplementary file 1 — Additional file 1: MRD assessment. [file 13287_2022_3186_MOESM1_ESM.docx]

**Supplemental data**

**MRD assessment**

**RT-qPCR for BCR/ABL fusion gene**

Mononuclear cells were separated from fresh bone marrow samples by Ficoll-Hypaque gradient centrifugation. Total RNA was extracted using Trizol Reagent (Invitrogen, Carlsbad, CA, USA) according to the manufacturer’s instructions.

Reverse transcription was performed using random hexamer primers (final concentration 5ng/μl; Promega, USA) and Moloney murine leukemia virus reverse transcriptase (final concentration 10U/μl; Promega, Madison, WI, USA) for total RNA (2μg/20μl).

TaqMan-based RQ-PCR technology was used. PCR reactions and fluorescence measurements were performed with an ABI PRISM 7500 real-time PCR system (PE Applied Biosystems, Foster City, CA,USA). We selected ABL as a control gene to compensate for variations in quality and quantity of RNA and cDNA. BCR-ABL primers and probe that amplified both e1a2 and b3a2 or b2a2 junctions were designed using Primer Express software version 5.0. Sequences were as follows:

e1a2 BCR/ABL:

Forward primer 5’-ATCGTGGGCGTCCGCAAGAC-3’

Reverse primer 5’-GCTCAAAGTCAGATGCTACTG-3’

Probe 5’-FAM-CGCCCTCGTCATCGTTGGGCCAGATCT-TAMRA-3’

b3a2 or b2a2 BCR/ABL:

Forward primer 5’-CCGCTGACCATCAATAAGGAA-3’

Reverse primer 5’-CTCAGACCCTGAGGCTCAAAGT-3’

Probe 5’-FAM-AGCCCTTCAGCGGCCAGTAGCATCT-TAMRA-3’

ABL primers and probe were referred to the report of the Europe Against Cancer Program[1]. The PCR reaction mixture contained 10μl of 2×TaqMan Universal PCR Master Mix (PE Applied Biosystems), each primer at a concentration of 300 nM,200 nM probe and 2μl of cDNA in a total volume of 20μl. PCR was performed under the following conditions: 50℃ for 2 min, then 95 ℃ for 10 min, followed by 40 cycles of 95 ℃ for 15 s and 62 ℃ for 1 min. Each PCR run included a negative control, a positive control and plasmids of serial dilutions.Following manufacturer’s suggestions, we employed one standard curve generated by a serial dilutions (10^6^-10^2^ copies) of plasmid containing ABL target sequence to quantitate both BCR-ABL and ABL copy numbers because of their similar PCR efficiency. PCR product was purified and cloned into pGEM-T vector.The copy number of the plasmid was calculated from the DNA concentration (determined by measuring A260) and the molecular weight of the plasmid. Any sample with <3×10^4^ copies of ABL was regarded as poor quality and was excluded from the test. Both BCR-ABL and ABL transcript of each sample were analyzed in duplicate. The BCR-ABL transcript level was calculated as BCR-ABL/ABL as a percentage and >10^-4^ was thought as positive.

References

1. Beillard E, Pallisgaard N, van der Velden VH, et al. Evaluation of candidate control genes for diagnosis and residual disease detection in leukemic patients using 'real-time' quantitative reverse-transcriptase polymerase chain reaction (RQ-PCR) - a Europe against cancer program. Leukemia. 2003;17(12):2474-2486.

**MRD assessment via flow cytometry**

Multiparametric flow cytometry was performed in all patients as a routine clinical

test on bone marrow aspirate samples that were obtained as part of the

baseline assessment before HSCT. Analyses were performed on a BD FACSCanto II with DIVA 7.0 software package. 0.2-1 million cells per tube were collected.The antibody panel included 2 combinations of the following anti-human monoclonal antibodies (BD Biosciences). One consisted of CD19, CD58, CD34, CD13+33, CD20, CD38 and CD45 (panel 1), coupled with the PE/CY7, FITC, PERCP-Cy5-5, APC, APC-Cy7, V450 and V500-C fluorochromes, respectively. Another contained CD19, TdT, CD22, CD34, CD81, CD10 and CD45 (panel 2), coupled with the V450, FITC, PE, PERCP-Cy5-5, PE-Cy7, APC and V500-C fluorochromes, respectively. The isotype control monoclonal antibodies were used. B cells were characterized by low SSC and CD19 expression. Considering the possible CD19 antigen escape after anti-CD19 CAR-T therapy, SSC/CD19 and SSC/CD22 were both used as the gating methods to identify B cells, to avoid false-negative results in post-CAR-T patients. CD34, CD10, CD45, CD38, CD20, TdT, and CD81 were used to further characterize these cells. Positive MRD was considered when more than 20 cells with leukemia-associated immunophenotypes (LAIP) and SSC characteristics, identified in all plots of interest and carrying at least two LAIP markers identified at diagnosis, were observed[1]. For those without LAIP markers at diagnosis, MRD was identified as a cell population showing deviation from normal patterns (DFNP) of antigen expression seen on specific cell lineages at specific stages of maturation compared with either normal or regenerating marrow. The standardized assays and quality controls were performed according to previous study[2]. When abnormal cells were identified, the cells were quantified as a percentage of the total CD45^+^ white cell events and >10^-4^ was thought as MRD positive (figure 1).


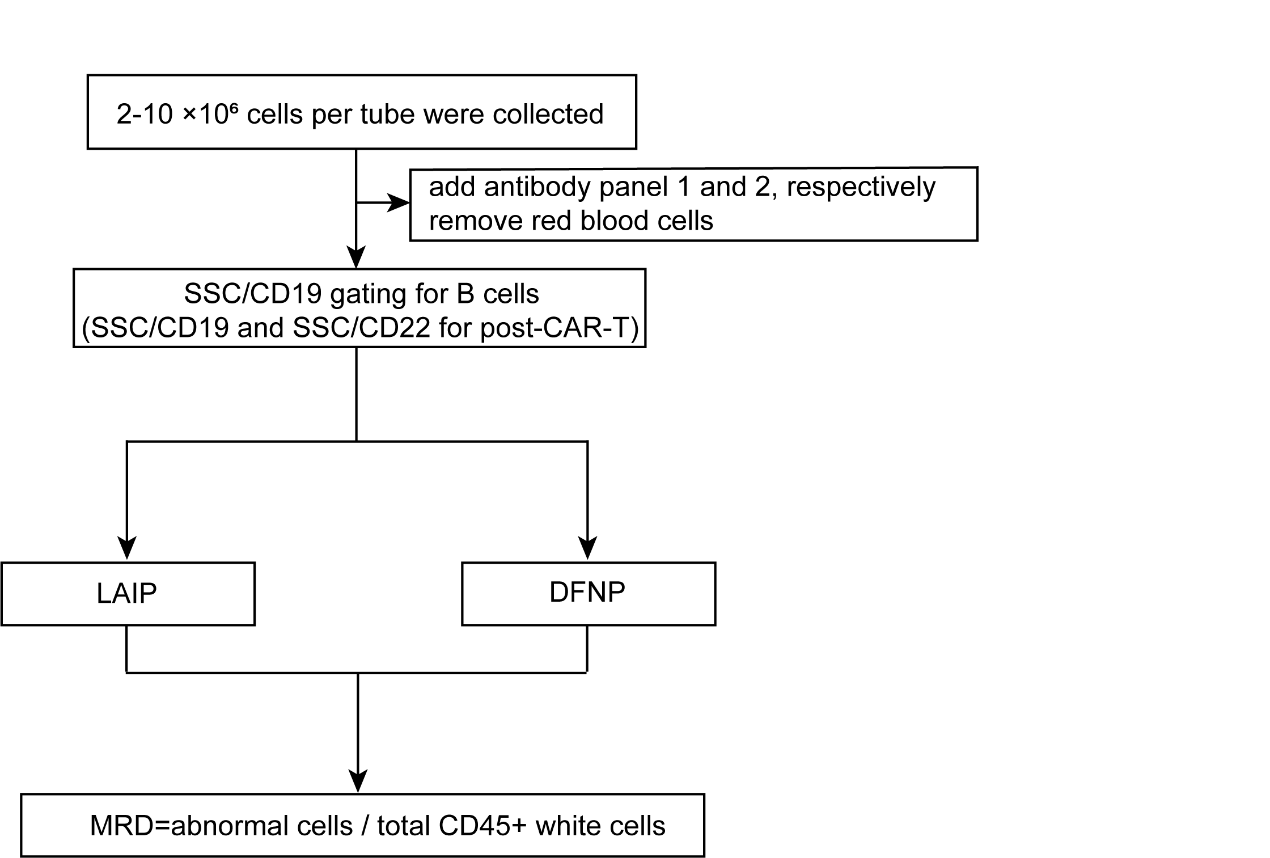


Figure 1. flow plot of MRD assessment via flow cytometry

References

1. Lucio P, Gaipa G, van Lochem EG, et al. BIOMED-I concerted action report: flow cytometric immunophenotyping of precursor B-ALL with standardized triple-stainings. BIOMED-1 Concerted Action Investigation of Minimal Residual Disease in Acute Leukemia: International Standardization and Clinical Evaluation. Leukemia. 2001;15(8):1185-1192.

2. Del Vecchio L, Brando B, Lanza F, et al. Recommended reporting format for flow cytometry diagnosis of acute leukemia. Haematologica. 2004;89(5):594-598.
